# What is the Price of Conservation? A Review of the Status Quo and Recommendations for Improving Cost Reporting

**DOI:** 10.1093/biosci/biac007

**Published:** 2022-03-23

**Authors:** Thomas B White, Silviu O Petrovan, Alec P Christie, Philip A Martin, William J Sutherland

**Affiliations:** Conservation Science Group, Department of Zoology, University of Cambridge, Cambridge, England, United Kingdom; Conservation Science Group, Department of Zoology, University of Cambridge, Cambridge, England, United Kingdom; Conservation Science Group, Department of Zoology, University of Cambridge, Cambridge, England, United Kingdom; Biosecurity Research Initiative, St Catherine's College, University of Cambridge; Downing College, Cambridge, England, United Kingdom; Conservation Science Group, Department of Zoology, University of Cambridge, Cambridge, England, United Kingdom; Basque Centre for Climate Change, Leioa, Spain; Conservation Science Group, Department of Zoology, University of Cambridge, Cambridge, England, United Kingdom; Biosecurity Research Initiative, St Catherine's College, University of Cambridge

**Keywords:** evidence-based conservation, effectiveness, efficiency, decision-making, cost-effectiveness

## Abstract

Wildlife conservation is severely limited by funding. Therefore, to maximize biodiversity outcomes, assessing financial costs of interventions is as important as assessing effectiveness. We reviewed the reporting of costs in studies testing the effectiveness of conservation interventions: 13.3% of the studies provided numeric costs, and 8.8% reported total costs. Even fewer studies broke down these totals into constituent costs, making it difficult to assess the relevance of costs to different contexts. Cost reporting differed between continents and the taxa or habitats targeted by interventions, with higher cost reporting in parts of the Global South. A further analysis of data focused on mammals identified that interventions related to agriculture, invasive species, transport, and residential development reported costs more frequently. We identify opportunities for conservationists to improve future practice through encouraging systematic reporting and collation of intervention costs, using economic evaluation tools, and increasing understanding and skills in finance and economics.

There have been repeated calls for conservation to move beyond decision-making based on anecdotal sources of information toward more informed processes based on the evidence of what does and does not work to improve biodiversity outcomes (Pullin and Knight 2001, Sutherland et al. 2004, Ferraro and Pattanayak 2006, Knight 2006). Much progress has been made on this, including the facilitation of freely accessible systematic reviews by the Collaboration for Environmental Evidence (CEE; www.environmentalevidence.org; Pullin and Knight 2009), and the development of the Conservation Evidence database (www.conservationevidence.com; Sutherland et al. 2019). Despite this progress, it is still common for decisions to be made without consulting evidence, even when such evidence may be readily available (Sutherland and Wordley 2017)—risking suboptimal biodiversity outcomes and wasted resources.

Even when evidence is available and included in decision-making, biodiversity conservation is chronically underfunded, and so financial constraints heavily rest on decision-makers and alter the set of outcomes that can be achieved (Miller et al. 2002, McCarthy et al. 2012). For example, the financial requirements for effective global biodiversity protection are estimated to be US$722 to US$967 billion a year, of which only US$124 billion to US$143 billion is received (Deutz et al. 2020). Therefore, in almost all situations, consideration of the financial costs of interventions is typically as important as assessing effectiveness if we are to optimize biodiversity outcomes. However, the measurement and reporting of costs in conservation has received much less attention than in other fields, such as healthcare, but its importance is increasingly being recognized (Cook et al. 2013). Conservationists have emphasized the significance of efficient resource allocation for the protection of species and habitats (Leader-Williams and Albon 1988), have shown that funding is not always directed according to conservation priority (Restani and Marzluff 2002), and have highlighted the extreme variation in costs of different conservation programs geographically (e.g., Balmford et al. 2003, Massei et al. 2010) and across different species and habitats (e.g., Laycock et al. 2011, Gordon et al. 2020). Studies have shown that including costs in assessments can massively improve the biodiversity returns possible on a given budget (Murdoch et al. 2007). This has been exemplified for a range of interventions, from systematic conservation planning (Ando et al. 1998, Naidoo et al. 2006, Field and Elphick 2019, Lessmann et al. 2019) to comparing interventions within and across species management programs (Laycock et al. 2011, Milner et al. 2014). In several cases, formal economic analyses have been used to assess the cost-effectiveness of programs—most often, cost-effectiveness analysis (Cullen et al. 2005, Gjertsen et al. 2014).

This realization of the importance of costs has led to calls to systematically report the costs of conservation interventions (Karesh 1993, Shwiff et al. 2013, Iacona et al. 2018, Murphy et al. 2021, Pienkowski et al. 2021). Doing this would aid explicit assessment of costs when selecting interventions (Squires and Garcia 2018, Booth et al. 2019) and would allow for the routine use of economic evaluation tools and cost-effectiveness frameworks (Metrick and Weitzman 1998, Hughey et al. 2003, Cook et al. 2017). Despite this progress, it seems likely that cost information is still rarely collected or reported when assessing effectiveness and is often not available to practitioners wanting to shift toward cost-effective practice (Grand et al. 2017). Recently, Pienkowski and colleagues (2021) showed that the number of studies assessing cost-effectiveness remains small, perhaps hindered by the challenges of standardized cost reporting. However, the scope of that review was relatively limited for the following reasons: it was restricted to studies that explicitly mentioned costs in their title and abstract, resulting in an underestimation of studies that discussed costs but whose main focus was on testing actions. And it did not detail the types of cost reported or include detail where partial costs were reported. Pienkowski and colleagues (2021) and previous research indicated that where cost information is reported, it is often aggregated, not standardized (Cook et al. 2017), and missing important costs relevant on the ground (Ban and Klein 2009, Robbins and Daniels 2012, Phelps et al. 2013). Without standardized costings, that clearly identify which costs are included in totals, it can be difficult to determine whether or not costs are complete or relevant to a given context.

## Reviewing the reporting of costs

Given the importance of cost information in decision-making, we investigated the reporting of cost information in published studies that quantitatively assess the effectiveness of conservation interventions to determine: (i) if costs are included in the discussion of the intervention's success or failure; (ii) if they are, what type of cost information is recorded; and (iii) how the reporting of costs changed over time, between geographies, types of interventions and targeted species or habitats.

By cost, we refer to the financial or monetary costs of a given conservation action, and by cost-effectiveness, we refer to the cost required to achieve a given unit of biodiversity change. To examine how often costs are reported and whether they are presented in a form that is useful for decision-making, we obtained English-language full texts of 1987 studies included within the Conservation Evidence database (Sutherland et al. 2019; www.conservationevidence.com) encompassing interventions to conserve a broad range of species and habitats as categorized into the Conservation Evidence synopses: shrubland and heathland (139 studies; Martin et al. 2017), peatland (149; Taylor et al. 2018), forests (274; Agra et al. 2016), terrestrial mammals excluding bats and primates (874; Littlewood et al. 2020), amphibians (317; Smith and Sutherland 2014), primates (64; Junker et al. 2017) and bats (157; Berthinussen et al. 2019), as well as 15 studies included in two or more of the above synopses. Each synopsis represents a systematically collated synthesis of the effectiveness of conservation interventions for that topic (Sutherland et al. 2019). Studies are included in the Conservation Evidence database based on systematic searches across over 650 peer-reviewed journals (including 30 major conservation journals), and selected gray literature (e.g., report series) using an approach known as subject-wide evidence synthesis (see the supplemental material; Sutherland et al. 2019). For each study, we collated information on geographical location and the date of publication. For papers in the mammal synopsis, we also collected information on the mammal species targeted and their IUCN Red List Categories (IUCN 2020), with each study being assigned to the highest Red List category represented by the species targeted by interventions in that study. The studies were also classified into different intervention types on the basis of IUCN classifications of direct threats and conservation actions (see the supplemental material for detailed descriptions).

A search string was then developed and tested to search within the full texts of the articles for cost information using a semiautomated approach to search within the study's full text (see the supplemental material). Where information on cost was identified in a document, the paragraph, figure, or table around each positive hit was read, along with the paper's abstract, to determine the level of cost reporting for the tested intervention. A full search methodology is provided in the supplemental material.

Table 1 lists the information extracted from the studies. The studies that reported costs were classified into five hierarchical levels of cost reporting, and the information on the types of cost were extracted. We calculated the proportion of assessed studies within each cost reporting category and then used logistic regression models to test whether the proportion of studies for a given category of cost reporting (1–5; table 1) differed significantly between synopses, publication dates, and study locations (see supplemental table S1). In addition, we conducted a separate statistical analysis for the mammal synopsis, in which we also tested for the effect of intervention type and the IUCN threat category of the species targeted (table S1). Further detail on statistical analyses and more detailed results from analyses are provided in the supplemental information.

**Table 1.** Cost information extracted from papers.

Type of information	Information	Description
Level of cost reporting	1. Mentioned cost	The study mentions the financial cost of the intervention, compares the intervention to the financial cost of an alternative or discusses the practicality of the intervention in relation to financial costs.
	2. Numeric cost	The study both mentions costs (as per Level 1) and also provides a numerical value of the cost for an intervention being tested or compared against
	3. Total cost	The study provides a total intervention cost reported. Where ambivalent, the total cost is assumed to be more than just the cost of consumables.
	4. State components	The study provides a total cost and states which costs are included or excluded in the total cost. These are broken down not just by activity, but into basic components such as labor, capital and consumables.
	5. Quantify components	The study provides a total cost, broken down into various components (as per Level 4) and quantifies how these components are broken down
If cost is reported, what types of costs are included? (Modified from Iacona et al. 2018, Adam and Murray 2003)	Direct Intervention Cost	The cost of the specific intervention, including labor, capital, and consumables.
	Labor	The cost of labor to conduct the intervention (e.g., hours required and wages for a given task).
	Capital	Cost of items required for the respective intervention (e.g., machinery, protective equipment, transportation equipment).
	Consumables	Cost of items used up during the intervention (e.g., fencing materials, herbicide, tree guards, educational materials).
	Overhead cost	Administration and management costs (e.g., planning costs, obtaining permits).
	Access cost	Cost of accessing the intervention (e.g., transport).
	Future cost	Future costs associated with the intervention (e.g., future management to control invasive species, required monitoring).
	Opportunity cost	The forgone income that could have been generated in an alternative scenario without the conservation action (e.g., loss of income from cropland).
	Avoided cost	The avoided cost as a result of an intervention (e.g., reduced cost of insurance claims because of fewer collisions, reduced cost of invasive species damage).
Additional information reported	Currency	Currency in which the cost was incurred.
	Date	Date when the cost was incurred.

a Where articles mentioned the cost of monitoring techniques for the study, this was only included if the monitoring was deemed necessary for the intervention and not just for the study.

## Poor and variable reporting of the costs of conservation interventions

**Figure 1.**
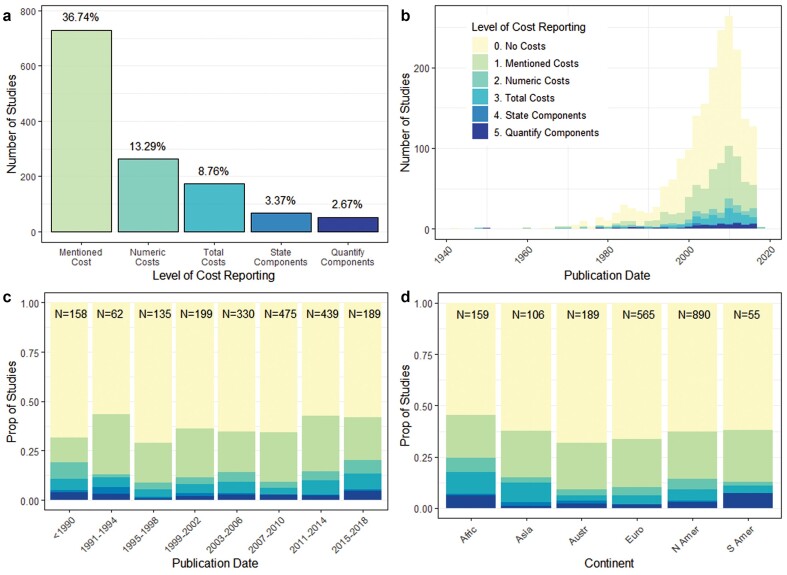
Level of cost reporting in studies that assess the effectiveness of interventions (n = 1987) split by (a) category of cost reporting, (b) publication date, (c) binned publication date and (d) continent. For each column in panels (c) and (d), the sample size is given at the top of the bar. In panels (a) and (b), the y-axis represents the number of studies; panel (b) shows the distribution of the data set over time. In panels (c) and (d), the y-axis displays the proportion of studies within each level of the explanatory variable. The distribution of studies shown in panel (b) is influenced by the publication dates of the different synopses included in the study that will have compiled literature up until different end years.

Cost reporting was infrequently undertaken in studies that tested the effectiveness of conservation interventions; only 36.8% of the studies mentioned the cost of interventions, and only 13.3% provided quantitative costs (figure 1a). Even within these studies, cost reporting was often not presented in a helpful form for determining cost-effectiveness because only 8.8% of the studies reported total costs, and even fewer (3.4%) stated or quantitatively reported (2.7%) the constituents of that total (figure 1a).

Cost reporting did not substantially increase over time for any of the numerical cost categories (p > .05; see table S1 and figure 1b–1c), although there was moderate evidence to suggest that newer studies mentioned the financial costs of interventions more frequently (eβ = 1.02, p = .002).

Pairwise comparisons provided strong evidence to suggest the proportions of studies reporting numeric and total costs were higher in Africa (numeric costs, .25; total costs, .18) compared with studies in Europe (numeric costs, .1; total costs, .06) and Australasia (numeric costs, .09; total costs, .06; p < .05, supplemental table S2). Although they were nonsignificant, weaker trends were identified between Europe and Africa at higher levels of cost reporting (p = .12 for stating the components of total costs; p = .11 for the quantifying components) and between the proportions of studies reporting total costs between Africa (.18) and North America (.09, p = .13). There was also weak evidence to suggest that South America had a higher proportion of studies providing detailed costings—that quantitatively reported the components of the total (.07)—than did studies in Australasia (.02, p = .14) and Europe (.02, p = .12; figure 1d, table S2). Finally, there was weak evidence to suggest North America had a higher proportion of studies mentioning costs (.37, p = .13), and reporting numeric costs (.14, p = .07), than those in Australasia (mentioning costs, .32; numeric costs, .09; figure 1d, table S2) although this trend diminished at higher levels of cost reporting (p = .46 when comparing studies that report total costs).

**Figure 2.**
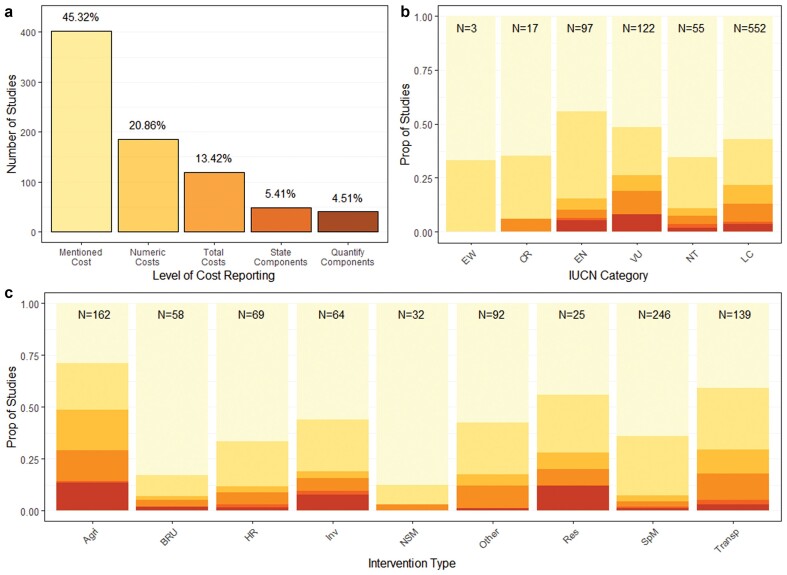
Level of cost reporting in mammal studies (n = 887) that assess the effectiveness of interventions split by (a) category of cost reporting, (b) IUCN category, and (c) intervention type. In panel (a), the y-axis represents the number of studies in each category. In panels (b) and (c), the y axis displays the proportion of studies within each level of the explanatory variable. For each column the sample size is given at the top of the bar. The bar colors in all graphs are consistent with the shading of columns in panel (a). Abbreviations: Agri, agriculture and aquaculture; BRU, biological resource use; CR, critically endangered; EN, endangered; EW, extinct in the wild; HR, habitat restoration; Inv, invasive and other problematic species, genes, and diseases; LC, of least concern; NSM, natural system modifications; NT, near threatened; Res, residential and commercial development; SpM, species management; Transp, transportation and service corridors; VU, vulnerable.

Our data provided strong evidence that the studies in the mammal synopsis had higher rates of reporting numeric costs than several other synopses, with 45.3% of the studies mentioning costs, 20.9% of the studies providing quantitative costs, 13.4% providing total costs, 5.4% stating components of that total, and 4.5% quantifying those components (figure 2a, table S2). There was also moderate evidence that the forest synopsis had a lower proportion of studies reporting numeric costs (.03) than the bat (.10), amphibian (.09), and mammal (.21) studies (p < .05; table S1), strong evidence that forest studies had a lower proportion reporting total costs (.02) and stating components of that total (less than .01) than did the mammal studies (total, .14; state components, .05, p < .05) and weaker evidence for the amphibian studies (total, .07, p = .11; table S2).

Within the mammal synopsis, the effect of publication date and geography were similar to that of the wider data set, although the North American studies had higher rates of cost reporting than did the European studies at multiple levels (p < .05; supplemental tables S3 and S4). We found large differences between the types of intervention (figure 2c, table S3); there was strong evidence that the studies focused on agricultural interventions had a higher proportion mentioning and reporting numeric costs (mentioned, .71; numeric, .49) than several other intervention types, including biological resource use (mentioned, .17; numeric, .06), habitat restoration (mentioned, .33; numeric, .12), species management (mentioned, .38; numeric, .07), and invasive species control (mentioned, .44, numeric, .19; p < .05; table S3) and a higher proportion of studies mentioning costs than interventions classed as natural system modifications (.13, p < .05; table S3). Similar evidence was identified at higher levels of cost reporting (see table S3). There was moderate evidence that studies covering residential and commercial development, transport, and invasive species management had a higher proportion of studies mentioning and reporting numeric costs than several other synopses (p < .05; table S3), whereas biological resource use, natural system modification, and species management had lower rates than several other synopses (p < .05; table S3). Across the analyses, there was little evidence for an effect of IUCN category on cost reporting (p > .05; figure 2b), apart from a higher proportion of studies mentioning costs when focused on species classified as Endangered than focused on Least Concern species (p = .01; table S3).

Of the studies that reached the highest levels of cost reporting (4 and 5), the majority reported on the costs of consumables used in the intervention (e.g., food provided for animals, herbicide, tree guards, fencing equipment) and labor costs (94% and 74%, respectively). Many studies also reported on the costs of some capital costs (37%), access costs (34%), and the costs of future management (29%). A smaller proportion of the studies reported avoided costs (19%), and the costs of administration (13%). No studies in the higher levels of cost reporting reported opportunity costs, although it is noted that these costs were reported in several studies at lower levels of reporting. All of the studies reported currency alongside the cost; 64% of the papers did not report dates explicitly with the costs.

## Implications for conservation

Despite the importance of costs in decision-making, we found that quantitative costs of interventions were only provided in 13.3% of the studies that tested the effectiveness of conservation interventions. Total costs were provided for 8.8% of the studies but with estimates often aggregated and missing costs often important in practice (e.g., overheads, capital, opportunity costs). However, we found that rates of cost reporting differed significantly between different regions, the habitat or species’ targeted by the interventions, and intervention types. Worryingly, we also found that the reporting of numerical costs has not increased over time, although the proportion of studies that mention costs without reporting them has increased. Without access to quantitative cost data, assessing the cost-effectiveness of conservation action is impeded.

We found that even when costs are reported, they often omit crucial information, including the date when the cost was incurred, and relatively few studies stated or quantified the components of total costs. These results support the findings of Iacona and colleagues (2018), who found that for 30 papers that reported costs of interventions the majority did not report detailed costs. We found that, when the components of costs were reported, they were most often the costs of direct implementation (e.g., consumables, labor), whereas the reporting of other cost types (e.g., admin, opportunity, capital) was variable. Because costs are highly context dependent, a lack of information on how total cost has been calculated or on specific values for certain types of cost can make it difficult to determine the relevance of the costs to a given context (Evans and Popova 2016). For example, if investigating the likely costs of a reintroduction program from an organizational perspective, decision-makers may require estimates of material and equipment costs, labor fees, administration expenses, or losses in income to local farmers associated with human–wildlife conflict. Alternatively, a practitioner may only be interested in the marginal costs of supplemental feeding as part of the reintroduction program, and only require information on consumables and labor. Without detail on how published costs are calculated, the value of the limited information available is reduced and detailed assessments of cost-effectiveness are hindered. More optimistically, we did identify 8.8% of the studies that reported the total costs of interventions. Many of these studies explicitly investigated the cost-effectiveness of action, demonstrating the utility of appropriate collation of financial costs and the benefits of calculating and comparing the cost-effectiveness of different actions for improving biodiversity outcomes (see box 1).

Box 1. **Examples of cost reporting and assessments of cost-effectiveness.**

**Costs of fencing for excluding coyotes, Montana, United States.** Matchett and colleagues (2013) found that electric fencing reduced coyote (Canis latrans) movements into areas supporting breeding populations of black footed ferrets (Mustela nigripes) in Montana, in the United States. To investigate the cost-effectiveness, they reported one-time costs for fence construction in their project area (including fencing, batteries, solar panels, all-terrain vehicle) at $34,376 ($4464 per km) and ongoing costs (including labor for setup and maintenance and fuel) at $4939 per year ($641 per km). All costs are in 2011 US dollars. Using a 10-year time horizon, and an estimated increase in juvenile ferret survival of 20%–30%, they calculated that the cost per ferret saved would be between $3600 and $5400. However, they also outline that if the vehicle and labor were not paid for, this may decrease to between $1700 and $2550 making it more cost-effective for the funding agency.

**Using guard dogs to reduce human–wildlife conflict, Patagonia, Argentina.** Gonzalez and colleagues (2012) investigated the effectiveness and costs of livestock guardian dogs for reducing goat deaths, and subsequent retaliatory killing of carnivores (e.g., cougar Puma concolor or culpeo fox Lycalopex culpaeus). All eight herders using the working dogs reported lower losses from carnivore predation and no carnivore killing. At the time of the study in 2012, the total annual cost to a herder to maintain a dog was US$183 including food, antiparasitic drugs, vaccinations, and fuel for veterinarian trips. They estimate this cost is only 7% of the average livestock capital losses due to predation (US$2446 per year) suggesting that their use could be cost-effective for reducing conflict, and financially beneficial for herders if used properly.

**Building fortified bomas to reduce predation by carnivores, Kenya.** Sutton and colleagues (2017) show that the creation of fortified bomas using chain-link fencing can reduce average household livestock losses from 0.96 animals per month (in a situation with traditional thorn-bush fences) to 0.35 animals per month. They present detailed costs of boma creation for fully fortified (US$890.13, 77,860 Kenyan shillings [KSH]), partially fortified (US$446.32, 35,040 KSH) and unfortified bomas ($11.43,1000 KSH) including equipment, labor and transportation. Annual maintenance cost of fortified bomas was estimated at 5000 KSH. Although unfortified bomas are less costly, they quantify how the greater effectiveness of fortified bomas leads to substantial avoided losses for livestock owners. Their cost–benefit analysis estimated a net present value for boma creation of $5899.93 over the boma's 5-year lifespan. These costs are in 2013 US dollars and Kenyan shillings, using an exchange rate of 87.47 and a discount rate of 12%.

Frustratingly, it is likely that, for many conservation interventions, someone is aware of the costs associated with it, and sometimes this information may be available outside of the published research literature. For example, there are databases on costs associated with invasive species management (Diagne et al. 2020), agricultural interventions (United Kingdom: Redman 2018; United States: Farm Bill Funding Costs, USDA 2021a), endangered species recovery plans (Miller et al. 2002), and project reports often outline the expenditure of a funded project. But for many conservation actions, such information does not make its way into the peer-reviewed or accessible gray literature and is not presented or collated alongside information on effectiveness—preventing others from benefiting from this knowledge and assessing efficiency. It is also possible that authors may detail costs more frequently in peer-reviewed studies that have been excluded from the Conservation Evidence database, such as those that only model the effect of an intervention or studies that solely report the costs of interventions but not their effectiveness. For example, there is a substantial body of work looking at the economic costs and benefits of payment for ecosystem service schemes (e.g., Zheng et al. 2013), but such work would not be included in the database.

Second, we excluded 34 studies contained in the Conservation Evidence database that were not written in English, potentially biasing the results of our review (Konno et al. 2020). Several synopses of taxa and habitats covered in our review (forests, shrublands, peatlands, and primates) did not consider non-English-language papers, meaning that our review will have underestimated the total number of papers in languages other than English. Despite this acknowledged bias in using only English-language studies, it is unlikely that adding the relatively small number of studies in other languages would have substantially altered the main conclusions of our study (Christie et al., 2020, 2021a).

## Reasons for trends in cost reporting

There are several possible explanations for the trends we identify in this study. We discuss these trends below.

### Low levels of cost reporting

Low levels of cost reporting have been found in more specific areas of conservation science and in other mission-driven scientific disciplines (table 2; Fischer and Lindenmayer 2000). For example, quantitative cost reporting in studies testing the effect of agrienvironment interventions on biodiversity is low (23%), although slightly higher than we found in this study—potentially because of the economic focus of these actions being linked to the loss of agricultural income and subsidy payments. In the humanitarian sector, a lack of an understanding of costs of interventions impedes cost-effectiveness assessments (Puett 2019). Reasons for the low rates of cost reporting may be similar across disciplines and include a lack of training in economics, low knowledge of economic assessment tools and their relevance to an organization, and a philosophical aversion to incorporating costs as a factor into decisions regarding humanitarian and environmental projects (Ansell et al. 2016, Grand et al. 2017, Puett 2019). In conservation, it has also been suggested there are limited incentives to move toward cost-effective practice because of low public pressure or a lack of evidence-based policy guiding decisions (Grand et al. 2017). Others have suggested that there are difficulties providing full cost accounts for complex conservation projects where costs accrue at various levels and on various time horizons (Pienkowski et al. 2021). For example, a given conservation action or project may be conducted over several years and may be funded by several different private foundations and government agencies, with financial costs and benefits accrued differently by different stakeholders, making it difficult to collate cost data for publication.

**Table 2.** Comparison of different reviews that have assessed the reporting of costs in conservation and other mission-driven disciplines (excluding medicine).

Source	Topic	Percentage of studies reporting numeric costs
Fischer and Lindenmayer 2000	Species reintroductions	3% provide numeric costs of interventions.
Ansell et al. 2016	Agrienvironment actions	48% mentioned costs, 23% provide numeric costs of interventions, 17.5% report total costs.
McEwan 2015	Learning in primary schools in developing countries	44% report some details on the cost of interventions.
Our study	Conservation interventions	37% mention costs, 13% provide numeric costs of interventions, 9% report total costs.

We found that the proportion of conservation intervention studies mentioning costs has increased slightly over time, offering hope that the importance of costs for practical conservation decision-making is being recognized in the scientific community, albeit slowly and insufficiently for generating a real change. These results concur with others who have shown that studies assessing cost-effectiveness in conservation are increasing slightly over time (Pienkowski et al. 2021) and that more recent agrienvironment studies were more likely to mention costs (although not to report them; Ansell et al. 2016). One potential explanation for this trend is that, in an increasing number of applied journals, it is now common to expect and discuss the practical implications and management considerations of research findings, perhaps encouraging authors to mention costs.

### Geography

Our results provide evidence that studies from Africa (strong evidence) and South America (weak evidence) have higher levels of detailed cost reporting. This appears to be in contrast with the findings of Pienkowski and colleagues (2021), who found a smaller number of studies of cost-effectiveness in these regions, although their focus on the number of studies rather than the proportion of all studies (as used in our study) makes comparison challenging. Given the large publication bias that typically exists toward the publication of conservation science research in North America, Europe, and Australasia (Christie et al., 2020, 2021a), only considering the total number of studies could mask patterns in how well costs are reported between different regions that publish different numbers of studies (Pienkowski et al. 2021). Therefore, the results of this study, alongside Pienkowski and colleagues (2021), suggest there may be more studies with detailed considerations of costs and cost-effectiveness in Europe and North America, but a proportionally greater reporting of costs in tropical regions of the world. Without investigating the specific projects and types of authors that were involved in tests, it is difficult to firmly assess why this may be the case. Possible reasons include: that costs are more often prohibitive for conservation interventions outside of Europe, Australasia, and North America, and so there is a greater focus on the costs of an intervention to assess its practicality. Secondly, it may be that practitioner-led tests of interventions are also more common in Africa and South America, as opposed to purely academic-led research, and that their costs are higher on authors’ agendas. Thirdly, it may be that many tests of interventions in Africa and South America are funded through specific grants and one-off conservation projects, which may be better costed in project proposals as opposed to ongoing projects.

### Type of intervention

Within studies of mammal conservation interventions, several types of intervention appear to be better costed, including interventions targeting invasive species control (e.g., herbicide application or manual removal), mitigating the effects of transport infrastructure (e.g., fences, underpasses, wildlife overpasses) and interventions on agricultural land (e.g., agrienvironment measures such as wildflower strips). Similarly, Pienkowski and colleagues (2021) found that invasive species control interventions were highly represented in studies that assessed cost-effectiveness in conservation, possibly because such interventions have clear spatial and temporal extents and easily measurable outcomes, making the assessments of their costs and effectiveness easier (Pienkowski et al. 2021). This subject also has a history of integrating financial considerations through agriculture and weed management (Pienkowski et al. 2021) and a stronger focus on the economics of interventions, including quantifying damage caused by invasive species (Diagne et al. 2020).

Our results identify that interventions related to activities whose goal may not primarily have been biodiversity conservation (e.g., transport, agriculture, residential development, invasive species control) reported costs more frequently than studies where conservation was a major goal (e.g., species management). There are several potential reasons for this difference. First, interventions such as invasive species control and those relating to transport infrastructure may often be motivated by reducing the financial costs associated with not doing them (e.g., a loss of crop yields due to invasive species, road collisions with mammals, and insurance claims). In these situations, the cost of doing nothing is clearer than for an intervention that is focused entirely on species conservation and make these interventions much more amenable to comparing costs and benefits in financial terms. Second, a major goal of projects such as road construction and housing developments is to minimize project costs, placing a greater emphasis on the costs of conservation activities. Third, actions targeting biodiversity conservation are more likely to be part of larger projects that can be split into multiple stages, with few one-off large costs, in contrast to large one-off projects, such as construction, where the calculation of costs may be simpler.

## Improving the reporting and use of cost data

Our results affirm previous claims that there is a need for better collation, reporting, and use of cost data in conservation in the published literature. On the basis of the trends observed above, we think that there are three major actions that could help improve the use of cost data for conservation decision-making: encouraging standardized reporting and collation of intervention costs, using economic evaluation tools, and building capacity in the conservation sector to address financial and economic issues. Together, these actions can ensure that cost data are available and are used appropriately and frequently in conservation decision-making to deliver potential efficiency gains. We detail these suggestions below and provide examples of how appropriate reporting of costs can improve conservation practice (box 1).

Standardized reporting of the costs of interventions using set frameworks can allow others to access costs, conduct economic analyses with the data, and determine the relevance of those costs to their circumstance. We add to the calls for the greater use of such standardized reporting frameworks that allow different types of costs to be recorded at various scales (e.g., intervention level, organization level) and alongside additional data, such as date and currency, that are required to make sense of the reported costs of conservation action (box 1; Cook et al. 2017, Iacona et al. 2018, Pienkowski et al. 2021). Frameworks exist for recording the direct costs of interventions alongside important contextual information (Iacona et al. 2018) and for measuring wider economic costs and benefits of action (Murphy et al. 2021). We also encourage journals to support and include the reporting of the costs in papers that test the effectiveness of interventions (as is already done by Conservation Biology).

Where detailed costings are required, such reporting should include not just the direct costs of implementation but also changes in future finances associated with the outcomes of the intervention (e.g., opportunity costs, avoided costs, future management and economic benefits), because these are vital considerations when assessing cost-effectiveness (see box 1). It is important to carefully define target and nontarget intervention outcomes, because what appears cost-effective for one measure of success may not always be for another. Resources exist that can help conservationists think through the likely types of costs and outcomes of different actions (e.g., Conservation Practice Effects sheets from the US Department of Agriculture; USDA 2021b). A given action may have multiple types of financial cost and different outcomes for environmental factors (e.g., biodiversity, water quality, soil retention), many of which could also be valued financially and included in assessments of costs and cost-effectiveness. The step-by-step framework developed by White and colleagues (https://osf.io/kd83v [preprint: not peer reviewed]) can be used to record the financial costs and benefits of conservation interventions (and estimate true economic cost), so that recorded figures can be used in economic analyses.

In addition, as more data on costs become available, we also encourage the compilation of the costs of interventions. For example, databases such as InvaCost have been established for the costs of invasive species interventions (Diagne et al. 2020). Wider data sets on the effectiveness of conservation interventions, including Conservation Evidence and the CEE, should also work to include costs in their syntheses (Pienkowski et al. 2021).

Standardized cost reporting and databases of collated costs would aid the use of economic evaluation tools in conservation, which can bring together information on effectiveness and costs, and help analyze the efficiency of conservation actions under a limited budget to reveal potential conservation efficiency gains (e.g., Cullen et al. 2005, Gjertsen et al. 2014, Morgans et al. 2019). Such evaluation tools, particularly cost-effectiveness analysis, are used frequently in other sectors including the military (Melese et al. 2015), education (Levin 1995), and healthcare industries (Adam and Murray 2003), and although they are increasingly used in conservation, their usage is the exception rather than the rule (box 1; Kubasiewicz et al. 2016, Pienkowski et al. 2021). Calculating and comparing cost-effectiveness requires information on the conservation effectiveness of actions to be available and directly comparable between interventions. Although there is no universally agreed metric for measuring effectiveness in conservation, databases and reviews of effectiveness information can be harnessed (e.g., Conservation Evidence, CEE), as can bespoke monitoring to obtain effectiveness data (e.g., see box 1). We encourage practitioners and academics to deploy these tools in conservation settings (Cook et al. 2017) while being vigilant that cost-effectiveness is not the only factor important for decision-making (Weidenfield et al. 2021) and that such analyses can mask important variation in the distribution of costs and benefits to different stakeholders (Waldron et al. 2020).

We also encourage the development and use of other decision-making tools for the assessment of cost-effectiveness that may be applicable even when detailed, relevant data on costs and effects may be lacking—as we have shown to often be the case—or where wider consideration of other factors (e.g., the distribution of financial costs and benefits, stakeholder values, acceptability of outcomes) are important (e.g., multi criteria decision analysis; Gerber et al. 2018, Knight et al. 2019). A major difficulty of scaling up cost-effectiveness tools is the lack of incentive for individuals and organizations to change their practices (Grand et al. 2017). Therefore, the increased use of these tools must be combined with the promotion of cost-effective practice in decision-making across organizations.

Finally, an insufficient knowledge of economics, evaluation tools, or issues of organizational culture and confidentiality can prevent costs from being collated, shared, and used (Grand et al. 2017), not allowing potential efficiency gains to be realized. Training and clear guidance on the importance of collecting standardized costs, how to report them, and how to use evaluation tools would build capacity in the conservation sector to address financial and economic issues. This could be combined with training on the use of specific software packages or tools that can help organizations report and assess cost-effectiveness and guidance materials on how to make evidence-based cost-effective decisions. For example, Christie and colleagues (2021b) provided a tool to aid decision-making about conservation actions, with costs and cost-effectiveness being an important step in their process that helps practitioners think through the likely biodiversity outcomes (i.e., effectiveness), financial costs (including cost effectiveness), acceptability and feasibility of different actions.

## Outlook

As we look to tackle biodiversity loss, it is vital that conservation interventions are efficient and make the best use of the limited funds available. However, limited reporting of costs will hamper this transition to more efficient practice. Researchers, editors, and practitioners should look to improve the reporting and collation of costs of conservation interventions, learning from areas of conservation and other sectors with higher consideration of costs. Doing so will allow conservationists to incorporate costs into decision-making—including through economic evaluation tools—ensuring the most cost-effective actions are selected to protect and restore biodiversity.

## Supplementary Material

biac007_Supplemental_File

## Author Biographical

Thomas B. White (tbw27@cam.ac.uk), Silviu O. Petrovan, Alec P. Christie, Philip A. Martin, and William J. Sutherland are members of the Conservation Science Group within the Department of Zoology at the University of Cambridge, in Cambridge, England, in the United Kingdom. APC and WJS are also affiliated with the Biosecurity Research Initiative at St Catherine's College, University of Cambridge. PAM is currently affiliated with the Basque Centre for Climate Change, in Leioa, Spain. APC is also affiliated with Downing College, in Cambridge, England, in the United Kingdom.
