# Supplementary material for: What is the Price of Conservation? A Review of the Status Quo and Recommendations for Improving Cost Reporting
Source: Bioscience. 2022 Mar 23;72(5):461–71. doi: 10.1093/biosci/biac007 (PMC9113343; doi:10.1093/biosci/biac007)
Supplement: biac007_Supplemental_File [file biac007_supplemental_file.docx]

**Supporting Information – Detailed Methodology**

We reviewed a total of 2166 studies included within the Conservation Evidence database (Sutherland et al. 2019; www.conservationevidence.com) encompassing interventions to conserve a broad range of species and habitats as categorised into the Conservation Evidence synopses: shrubland and heathland (Martin et al. 2017), peatland (Taylor et al. 2018), forests (Agra et al. 2016), terrestrial mammals excluding bats and primates (Littlewood et al. 2020), amphibians (Smith & Sutherland 2014), primates (Junker et al. 2017) and bats (Berthinussen et al. 2019). Papers on farmland measures were not included as a similar study has already been conducted for this topic (Ansell et al. 2016). Each synopsis represents a systematically collated synthesis of the effectiveness of conservation interventions for that topic (Sutherland et al. 2019). From these synopses we obtained English full-text copies of 1987 studies (317 amphibian studies, 157 bat studies, 274 forest studies, 874 terrestrial mammal studies, 149 peatland studies, 64 primate studies, 137 shrubland and heathland studies, and 15 studies that were included in multiple synopses). Papers that were written in non-English languages (36 studies) or where the full text could not be accessed (143 studies) were not included in this review.

Studies are included in the database based on systematic searches across hundreds of peer-reviewed journals (including 30 major conservation journals), and selected grey literature e.g., report series using a process known as subject-wide evidence synthesis. The titles and abstracts of all papers in journals and years of publications searched are screened to see if they are testing the effectiveness of a conservation intervention. If they are, the study is assigned for inclusion in the database. The full study is then summarised into a short paragraph, and assigned to the action (or actions) that the study is testing the effectiveness of. The full database is available online, with a detailed description and methodology published by Sutherland et al (2019). The database is therefore a subset of the literature that tests the effectiveness of conservation actions and does not include wider studies in conservation science or some other sources of non-academic literature (e.g., such as project reports, or guidance documents).

For each study, we collated information on year of publication, and geographical location of the study by assigning each study to a country (or countries), and continent. Twenty-three studies were conducted globally (e.g., reviews of studies across geographies) or in countries spanning multiple continents and were not assigned to a continent.

For papers in the mammal synopsis, we also collected information on the mammal species targeted by that intervention and the type of intervention tested. Mammal species assigned to each study were downloaded from the Conservation Evidence database, with studies not assigned to specific species being manually checked to ensure all studies looking at the effects of interventions on specific mammal species were tagged appropriately. We then assigned each species to an IUCN Red List Threat category (EW = Extinct in the Wild, CR = Critically Endangered, EN = Endangered, VU = Vulnerable, NT = Near Threatened, LC = Least Concern, DD = Data Deficient), using data downloaded from the IUCN Red List (2021). Each study was assigned to the highest threat category covered by species targeted by that intervention. Forty-one studies were not assigned to an IUCN threat category, as they either targeted groups/communities of mammal species or were only tagged with domestic or feral species not classified in the IUCN Red List.

To categorise studies into types of intervention tested, we used the chapter structure used in the terrestrial mammal synopsis (Littlewood et al, 2020) which separates different actions into chapters based on how they address threatening processes or restore species/habitats, in accordance with the IUCN threat and action categories. Descriptions of each category are given in Table S0, with all actions within this category presented by Littlewood et al (2020).

***Table S0: Intervention types as defined in Littlewood et al (2020).***

| Chapter | Definition / Interventions included (modified from Littlewood et al 2020) |
| --- | --- |
| Agriculture & Aquaculture | Agriculture and aquaculture can threaten mammal populations, largely though land use change and pollution. This chapter includes interventions specific to reducing the impacts of agricultural intensification in farmland, or aquaculture in seascapes, on biodiversity. Interventions include e.g.: ‘establish wildflower areas on farmland’, ‘manage hedgerows to benefit wildlife on farmland’ and ‘plant trees on farmland’. |
| Biological Resource Use | The use of biological resources for food, production, sport or recreation can threaten mammal species either directly (e.g., hunting) or indirectly (through logging). This chapter includes measures that restrict or minimize the effects of biological resource use. Specific interventions include: ‘prohibit or restrict hunting of a species’, ‘incentivise species protection through licensed trophy hunting’ and ‘use selective harvesting instead of clearcutting’. |
| Climate Change & severe weather | Climate change represents a long-term threat to many mammal species through e.g., changing habitat availability. This chapter includes interventions specific to addressing this threat and includes actions such as ‘protect habitat along elevational gradients’, and ‘provide dams/water holes during drought’. |
| Education & Awareness | This chapter includes conservation interventions aimed specifically at education campaigns, awareness raising and public engagement are described in this chapter. |
| Energy Production & Mining | Energy infrastructure (renewable and non-renewable) and mining can impact mammal populations through the destruction of habitats and pollution. This chapter includes interventions that specifically aim to tackle these threats. Specific interventions include: ‘restore former mining sites’ and ‘use electric fencing to deter mammals from energy installations’. |
| Habitat Protection | This chapter includes actions used to protect mammal habitats. Interventions include: ‘legally protect habitat for mammals’, ‘increase size of protected area’ and ‘build fences around protected areas’. |
| Habitat Restoration | This chapter includes interventions used to restore areas of habitat for mammal species. Interventions include e.g.: ‘restore of create grassland’, ‘restore or create wetlands’ and ‘provide artificial refuges/breeding sites’. |
| Human Intrusion & Disturbance | Threats to mammals can come from human intrusion and disturbance of habitats, in addition to large scale disturbance from agriculture, development and energy production. This chapter includes interventions to manage such disturbance and wildlife conflict. Specific interventions include: ‘provide paths to limit extent of disturbance to mammals’, ‘set minimum distances for approaching mammals’ and ‘use non-lethal methods to deter carnivores from attacking humans’. |
| Invasive & other problematic species | Invasive species, alien species and diseases can be problematic for some mammal populations. This chapter includes interventions specific for managing these problematic species. Interventions include e.g.: ‘use fencing to exclude grazers or other problematic species’, ‘remove/control non-native mammals’, and ‘remove or control predators’. |
| Multiple types of intervention | Studies that were included in multiple chapters were classified under this category. This means that a study tested a diverse range of interventions. |
| Natural System Modifications | Threats to mammals can be caused through the management or natural processes that occur in natural or semi-natural systems (e.g., fires, water flow). This chapter includes measures to address these threats. Specific interventions include e.g.: ‘use prescribed burning’, and ‘remove understory vegetation in forest’. |
| Pollution | Pollution can have direct and indirect threats on mammal populations e.g., poisoning of mammals, and degradation of wetland habitats. This chapter includes interventions specific to minimizing the impact of pollutants on mammal populations. Specific interventions include: ‘reduce pesticide or fertilizer use’ and ‘translocate mammals away from site contaminated by oil spill’. |
| Residential & commercial development | Residential and commercial developments can causing the destruction of habitat and pollution, impacting mammal populations. This chapter includes interventions specific to addressing the threats from residential and commercial development. Interventions include e.g.: keeping domestic animals indoors to reduce mammal deaths, preventing animals reaching waste or food sites in residential developments, or retaining wildlife corridors in residential developments. |
| Species management | Interventions not addressed at single threats but aimed specifically at increasing the populations of mammal species. Interventions include e.g., ex-situ conservation, translocations and providing artificial dens. |
| Transportation & service corridors | Transport infrastructure can cause impacts on mammal populations through land use change and human-wildlife conflict/collisions. This chapter includes interventions specific to addressing the threats from transportation infrastructure (e.g., road vehicles, trains) and service corridor construction and use. Specific interventions include: ‘install tunnels/culverts under roads’, ‘reduce legal speed limits’, and ‘install barrier fencing along roads and railways’. |

A search string was then developed and tested to search within the full texts of the articles for cost information:

*cost OR pay OR expen OR value OR cheap OR pound OR dollar OR price OR mone OR € OR £ OR $ OR gbp OR usd OR eur.*

A semi-automated approach was used to search the PDFs using Adobe Acrobats’ Advanced Search Function where the full text was searched for all search terms. Each positive hit was then screened by a single reader who read the sentence around the search term to determine if the text was discussing financial cost. Where information on cost was identified in a document, the paragraph or figure/table around each positive hit was read along the paper’s abstract to determine the level of cost reporting for the tested intervention. The full document was examined for PDFs that returned no hits and had corrupted text overlays.

*Extracting data:*

Table 1 lists the information extracted from the studies. Studies that reported costs were classified into five hierarchical levels of cost reporting. Where numerical cost information was reported, information on type of cost, and associated information was collected including a series of yes/no questions of reporting category and type.

*Testing the search methodology:*

The search terms and methods were tested using papers known to contain cost information. From Ansell et al (2016) we randomly selected ten papers mentioning economic cost, and ten papers providing numeric costs of farmland interventions. We also selected ten papers testing terrestrial mammal conservation interventions (randomly selected from those flagged as containing cost information by the authors of Littlewood et al. (2020)); and two papers on bird conservation identified as reporting costs during the current update of the bird synopsis (Williams et al. 2013). These were searched using the methodology above; in all cases, the mention, or reporting of costs was correctly identified.

To test whether our search terms were preferentially selecting costs from some countries over others, we tested a search string including the three letter currency abbreviations and written form of the currency from the country the study was conducted on papers in the primate synopsis – this synopsis contains very few studies in Europe or North America. We found that all those that reported costs would have been included without the country-specific search term as they reported costs intervention costs in USD or GBP.

*Analysis:*

We calculated the proportion of assessed studies within each cost reporting category. Using logistic regression models, we tested whether the proportion of studies for a given category of cost reporting (1-5; Table 1) differed significantly between synopses, publication dates and study locations (see Supporting Information – Table S1). We used binomial logistic regression models because the data violated the parallel trends assumption for an ordinal regression. In addition, we conducted a separate statistical analysis for the mammal synopsis, in which we also tested for the effect of intervention type and the IUCN threat category of the species targeted (Table S1). Where multiple species were targeted, the species with the highest level of threat was used to assign threat category. For this analysis, intervention types which contained less than 20 studies (climate change, education & awareness raising, energy production and mining, habitat protection, human intrusion and disturbance, pollution) and studies targeting multiple types of intervention were reclassified as ‘Other intervention types’ to ensure that sample size within each level was large enough to conduct the analysis. This is the classification presented in Figure 2 in the main text, but the full split of studies into intervention types is presented in Figure S1.

Where explanatory factors had more than two levels, Tukey pairwise comparisons were used to test for significance between levels. Analyses were conducted in R (R Core Team, 2018), using the *emmeans* package for conducting pairwise comparisons between levels of the explanatory factors. P values are corrected in this package to account for the multiple comparisons.

Table 1 is included in the main manuscript.

**Supporting Information – Tables & Figures**

| ***Table S1: Binomial logistic regression coefficients showing the effect of publication date on the proportion of studies reporting costs for each category of cost reporting.*** | | | | | | | | |
| --- | --- | --- | --- | --- | --- | --- | --- | --- |
| **Response Variable** | **β** | | **SE(β)** | **Odds Ratio (e^β^)** | **Upper CI** | **Lower CI** | **Z** | **P Value** |
| 1 Mentioned (Y / N) | | 0.017 | 0.005 | 1.017 | 1.028 | 1.006 | 3.053 | 0.002* |
| 2 Numeric (Y / N) | | 0.004 | 0.007 | 1.004 | 1.018 | 0.990 | 0.503 | 0.615 |
| 3 Total (Y / N) | | 0.004 | 0.009 | 1.004 | 1.021 | 0.988 | 0.494 | 0.621 |
| 4 Stated (Y / N) | | -0.004 | 0.013 | 0.996 | 1.022 | 0.972 | -0.288 | 0.773 |
| 5 Quantified (Y / N) | | 0.006 | 0.015 | 1.006 | 1.036 | 0.976 | 0.366 | 0.714 |
|  | |  |  |  |  |  |  |  |

*Significant effect detected P<0.05

***Table S2: Results of pairwise comparisons of the proportion of studies reporting costs between conservation topics and geographies. This table shows only comparisons between levels where P<0.15 to limit the size of the table and highlight the most important results ranging from weak to strong evidence of effects. All other comparisons between levels of the predictor variables (show in Fig 1) had a P>0.15.***

| **Response** | **Predictor** | **Comparison between types of intervention.** | **B** | **SE** | **Odds Ratio (exp(B))** | **Upper CI** | **Lower CI** | **Z** | **Adj P*** |
| --- | --- | --- | --- | --- | --- | --- | --- | --- | --- |
| 1 Mentioned (Y / N) | Geography | Africa - Australasia | 0.60 | 0.24 | 1.83 | 2.92 | 1.14 | 2.53 | 0.12 |
|  |  | Australasia - North America | -0.44 | 0.18 | 0.65 | 0.91 | 0.46 | -2.47 | 0.13 |
|  | Synopsis | Amphibian - Mammal | -0.83 | 0.15 | 0.44 | 0.58 | 0.33 | -5.60 | <0.001 |
|  |  | Bat - Mammal | -0.73 | 0.19 | 0.48 | 0.70 | 0.33 | -3.77 | <0.01 |
|  |  | Forest - Mammal | -0.84 | 0.16 | 0.43 | 0.58 | 0.32 | -5.46 | <0.0001 |
|  |  | Mammal - Peatland | 0.53 | 0.20 | 1.69 | 2.48 | 1.15 | 2.68 | 0.13 |
| 2. Numeric (Y / N) | Geography | Africa - Australasia | 1.30 | 0.33 | 3.68 | 7.03 | 1.93 | 3.95 | <0.01 |
|  |  | Africa - Europe | 0.82 | 0.26 | 2.28 | 3.76 | 1.38 | 3.24 | 0.02 |
|  |  | Australasia - North America | -0.76 | 0.28 | 0.47 | 0.81 | 0.27 | -2.72 | 0.07 |
|  | Synopsis | Amphibian - Forest | 1.29 | 0.41 | 3.62 | 8.11 | 1.62 | 3.13 | 0.04 |
|  |  | Amphibian - Mammal | -0.95 | 0.22 | 0.39 | 0.60 | 0.25 | -4.34 | <0.001 |
|  |  | Bat - Forest | 1.45 | 0.45 | 4.27 | 10.30 | 1.77 | 3.23 | 0.03 |
|  |  | Bat - Mammal | -0.78 | 0.28 | 0.46 | 0.80 | 0.26 | -2.77 | 0.10 |
|  |  | Forest - Mammal | -2.24 | 0.37 | 0.11 | 0.22 | 0.05 | -6.03 | <0.001 |
|  |  | Mammal - Peatland | 1.35 | 0.36 | 3.84 | 7.81 | 1.89 | 3.71 | <0.01 |
|  |  | Mammal - Shrubland & Heathland | 1.36 | 0.36 | 3.89 | 7.88 | 1.92 | 3.76 | 0.0042 |
| 3. Total (Y / N) | Geography | Africa - Australasia | 1.24 | 0.38 | 3.46 | 7.28 | 1.65 | 3.28 | 0.01 |
|  |  | Africa - Europe | 1.01 | 0.30 | 2.75 | 4.93 | 1.54 | 3.41 | 0.01 |
|  |  | Africa - North America | 0.65 | 0.26 | 1.92 | 3.22 | 1.14 | 2.47 | 0.13 |
|  | Synopsis | Amphibian - Forest | 1.30 | 0.47 | 3.68 | 9.27 | 1.46 | 2.76 | 0.11 |
|  |  | Amphibian - Mammal | -0.69 | 0.25 | 0.50 | 0.82 | 0.31 | -2.75 | 0.11 |
|  |  | Bat - Mammal | -1.09 | 0.41 | 0.34 | 0.74 | 0.15 | -2.69 | 0.13 |
|  |  | Forest - Mammal | -2.00 | 0.43 | 0.14 | 0.31 | 0.06 | -4.67 | <0.001 |
|  |  | Mammal - Shrubland & Heathland | 1.19 | 0.44 | 3.28 | 7.73 | 1.39 | 2.72 | 0.12 |
| 4. Stated (Y / N) | Geography | Africa - Europe | 1.17 | 0.46 | 3.22 | 7.99 | 1.30 | 2.53 | 0.12 |
|  | Synopsis | Forest - Mammal | -2.78 | 1.02 | 0.06 | 0.46 | 0.01 | -2.73 | 0.11 |
| 5. Quantified (Y / N) | Geography | Australasia - South America | -1.82 | 0.74 | 0.16 | 0.69 | 0.04 | -2.45 | 0.14 |
|  |  | Europe - South America | -1.61 | 0.64 | 0.20 | 0.70 | 0.06 | -2.51 | 0.12 |
|  |  | Africa - Europe | 1.22 | 0.47 | 3.38 | 8.55 | 1.33 | 2.57 | 0.11 |

*P value adjusted to account for multiple testing.

***Table S3: Results of pairwise comparisons of the proportion of mammal studies reporting costs between geographies, IUCN category, and type of intervention. The table is limited to comparisons where P<0.15 to limit the size of the table and highlight the most important results ranging from weak to strong evidence of effects. All other comparisons between levels of the predictor variables (shown in Fig 2) had P > 0.15.***

| **Response** | **Predictor** | **Comparison between types of intervention.** | **Β** | **SE(β)** | **Odds Ratio (e^β^)** | **Upper CI** | **Lower CI** | **Z** | **Adj P*** |
| --- | --- | --- | --- | --- | --- | --- | --- | --- | --- |
| 1 Mentioned (Y / N) | Type of Intervention | Agriculture - Biological resource use | 3.01 | 0.45 | 20.21 | 49.21 | 8.30 | 6.62 | <0.0001 |
|  |  | Agriculture - Habitat restoration & creation | 1.51 | 0.32 | 4.54 | 8.49 | 2.43 | 4.73 | <0.001 |
|  |  | Agriculture - Invasives | 1.17 | 0.33 | 3.24 | 6.19 | 1.69 | 3.55 | 0.01 |
|  |  | Agriculture - Natural system modifications | 3.28 | 0.64 | 26.63 | 92.83 | 7.64 | 5.15 | <0.0001 |
|  |  | Agriculture – Other | 1.33 | 0.30 | 3.76 | 6.71 | 2.11 | 4.49 | <0.01 |
|  |  | Agriculture - Species management | 1.56 | 0.23 | 4.75 | 7.48 | 3.01 | 6.72 | <0.0001 |
|  |  | Biological resource use - Habitat restoration & creation | -1.49 | 0.49 | 0.22 | 0.59 | 0.09 | -3.04 | 0.06 |
|  |  | Biological resource use - Invasives | -1.83 | 0.50 | 0.16 | 0.42 | 0.06 | -3.69 | 0.01 |
|  |  | Biological resource use - Other | -1.68 | 0.48 | 0.19 | 0.47 | 0.07 | -3.54 | 0.01 |
|  |  | Biological resource use - Residential & commercial development | -2.20 | 0.58 | 0.11 | 0.35 | 0.04 | -3.80 | <0.01 |
|  |  | Biological resource use - species management | -1.45 | 0.44 | 0.23 | 0.56 | 0.10 | -3.30 | 0.03 |
|  |  | Biological resource use - Transport | -2.26 | 0.46 | 0.10 | 0.25 | 0.04 | -4.97 | <0.0001 |
|  |  | Invasives - Natural system modifications | 2.11 | 0.67 | 8.23 | 30.52 | 2.22 | 3.15 | 0.04 |
|  |  | Natural system modifications - Other | -1.96 | 0.65 | 0.14 | 0.51 | 0.04 | -2.99 | 0.07 |
|  |  | Natural system modifications - Residential & commercial development | -2.47 | 0.73 | 0.08 | 0.36 | 0.02 | -3.37 | 0.02 |
|  |  | Natural systems modifications - Transport | -2.54 | 0.64 | 0.08 | 0.28 | 0.02 | -3.97 | <0.01 |
|  |  | Species management - transport | -0.82 | 0.24 | 0.44 | 0.71 | 0.28 | -3.40 | 0.02 |
|  | Geography | Europe - North America | -0.69 | 0.21 | 0.50 | 0.75 | 0.33 | -3.68 | 0.01 |
|  |  | Australasia - North America | -0.74 | 0.27 | 0.48 | 0.80 | 0.28 | -2.78 | 0.06 |
|  | IUCN | EN – LC | 0.86 | 0.26 | 2.37 | 3.92 | 1.44 | 3.38 | 0.01 |
| 2. Numeric (Y / N) | Type of Intervention | Agriculture - Biological resource use | 3.35 | 0.75 | 28.36 | 122.62 | 6.56 | 4.48 | <0.001 |
|  |  | Agriculture – Habitat restoration & creation | 1.86 | 0.42 | 6.42 | 14.56 | 2.83 | 4.45 | <0.001 |
|  |  | Agriculture – invasives | 1.23 | 0.39 | 3.43 | 7.32 | 1.61 | 3.19 | 0.04 |
|  |  | Agriculture – other | 1.64 | 0.35 | 5.13 | 10.24 | 2.58 | 4.65 | <0.01 |
|  |  | Agriculture - Species management | 2.41 | 0.30 | 11.15 | 20.18 | 6.15 | 7.95 | <0.0001 |
|  |  | Biological resource use - Residential & commercial development | -2.33 | 0.85 | 0.10 | 0.52 | 0.02 | -2.74 | 0.14 |
|  |  | Biological resource use - Transport | -2.53 | 0.76 | 0.08 | 0.35 | 0.02 | -3.35 | 0.02 |
|  |  | Invasives - Species management | 1.18 | 0.42 | 3.25 | 7.39 | 1.43 | 2.81 | 0.11 |
|  |  | Species management – transport | -1.59 | 0.33 | 0.20 | 0.39 | 0.11 | -4.79 | <0.001 |
|  | Geography | Africa - Europe | 1.11 | 0.43 | 3.03 | 6.99 | 1.32 | 2.60 | 0.10 |
|  |  | Europe - North America | -0.78 | 0.27 | 0.46 | 0.78 | 0.27 | -2.90 | 0.04 |
| 3. Total | Type of Intervention | Agriculture - Biological resource use | 2.52 | 0.75 | 12.38 | 54.17 | 2.83 | 3.34 | 0.02 |
|  |  | Agriculture – Other | 1.35 | 0.42 | 3.87 | 8.86 | 1.69 | 3.21 | 0.04 |
|  |  | Agriculture - Species management | 2.08 | 0.37 | 7.98 | 16.41 | 3.88 | 5.64 | <0.0001 |
|  |  | Agriculture - Habitat restoration & creation | 1.28 | 0.48 | 3.61 | 9.15 | 1.42 | 2.70 | 0.15 |
|  |  | Invasives - Species management | 1.53 | 0.48 | 4.60 | 11.88 | 1.78 | 3.15 | 0.04 |
|  |  | Species management - Transport | -1.59 | 0.41 | 0.20 | 0.45 | 0.09 | -3.90 | <0.01 |
|  | Geography | Africa - Europe | 1.51 | 0.51 | 4.54 | 12.22 | 1.69 | 3.00 | 0.03 |
|  |  | Europe - North America | -1.19 | 0.35 | 0.30 | 0.61 | 0.15 | -3.38 | 0.01 |
| 4. Stated | Type of Intervention | Agriculture - Species management | 2.02 | 0.53 | 7.52 | 21.43 | 2.64 | 3.78 | <0.01 |
| 5. Quantified | Type of Intervention | Agriculture - Species management | 2.46 | 0.66 | 11.73 | 42.68 | 3.22 | 3.73 | <0.01 |
|  |  | Agriculture - Transport | 2.11 | 0.77 | 8.24 | 37.51 | 1.81 | 2.73 | 0.14 |

*P value adjusted to account for multiple testing

| ***Table S4: Binomial logistic regression coefficients showing the effect of publication date on the proportion of mammal studies reporting costs for each category of cost reporting.*** | | | | | | | | |
| --- | --- | --- | --- | --- | --- | --- | --- | --- |
| **Response variable** | **B** | | **SE** | **Odds Ratio (exp(B))** | **Upper CI** | **Lower CI** | **Z** | **Adj P*** |
| 1 Mentioned (Y / N) | | 0.018 | 0.008 | 1.018 | 1.033 | 1.003 | 2.363 | 0.018 |
| 2 Numeric (Y / N) | | 0.012 | 0.009 | 1.012 | 1.030 | 0.993 | 1.113 | 0.210 |
| 3 Total (Y / N) | | 0.014 | 0.011 | 1.014 | 1.036 | 0.993 | 1.305 | 0.192 |
| 4 Stated (Y / N) | | 0.003 | 0.015 | 1.003 | 1.033 | 0.973 | 0.170 | 0.865 |
| 5 Quantified (Y / N) | | 0.015 | 0.018 | 1.015 | 1.052 | 0.980 | 0.840 | 0.401 |
|  | |  |  |  |  |  |  |  |

*Significant effect detected P<0.05


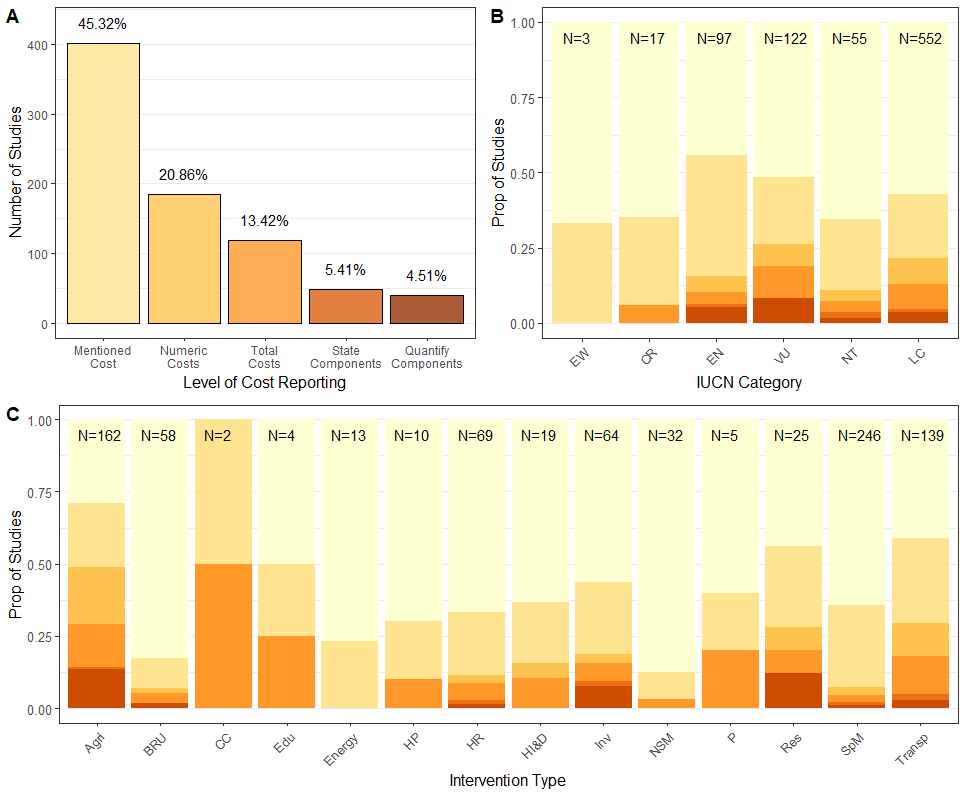


**Figure S1: Level of cost reporting in mammal studies (N = 887) that assess the effectiveness of interventions split by (A) category of cost reporting, (B) IUCN category^1^, and (C) intervention type^[[1]](#footnote-1)^. In (A) the y axis represents the number of studies in each category. In (B) and (C), the y axis displays the proportion of studies within each level of the explanatory variable. For each column the sample size is given at the top of the bar. Bar colours in all graphs are consistent with the yellow shading of columns in (A). This graph presents the data split by all intervention types, apart from studies classified as ‘multiple types of intervention’. For the analysis, and as presented in the main manuscript, ‘intervention types’ with N<20 were grouped into an ‘other’ category. Note that studies in multiple chapters have been excluded from 1C (N = 39), and for the statistical analysis all intervention type levels with N<20 were reclassified as ‘Other’ intervention types.**

1. Intervention type : Agri = Agriculture and Aquaculture, BRU = Biological Resource Use, CC = Climate Change, Edu = Education & Awareness, Energy = Energy production and mining, HP = Habitat protection, HR = Habitat restoration, HI&D = Human intrusion and disturbance, Inv = Invasive & other problematic species, genes and diseases,, NSM = Natural System Modifications, P = Pollution, Res = Residential and commercial development, SpM = Species Management, Transp = Transportation and Service Corridors.
   IUCN: EW = Extinct in the Wild, CR = Critically Endangered, EN = Endangered, VU = Vulnerable, NT = Near Threatened, LC = Least Concern. [↑](#footnote-ref-1)
